# Releasing the brake on plasma membrane H^+^-ATPases alters systemic defense

**DOI:** 10.1093/plphys/kiag595

**Published:** 2026-08-13

**Authors:** Yiwen Chen, Bo Xu

**Affiliations:** ARC Centre of Excellence in Plants for Space, and School of Agriculture, Food and Wine, Waite Research Institute, Adelaide University, Glen Osmond, SA 5064, Australia; ARC Centre of Excellence in Plants for Space, and School of Agriculture, Food and Wine, Waite Research Institute, Adelaide University, Glen Osmond, SA 5064, Australia; Assistant Features Editor, Plant Physiology, American Society of Plant Biologists

In plants, plasma membrane proton (H^+^)-ATPases are electrogenic P-type H^+^ pumps that couple ATP hydrolysis to active extrusion of H^+^ from the cytosol into the extracellular space (Pedersen et al. 2007). This activity modulates cytosolic and apoplastic pH, generates a transmembrane electrochemical H^+^ gradient, and controls membrane potential (*E*_m_) of plant cells, thereby providing the driving force for transmembrane solute transport and influencing electrical signal propagation (Farmer et al. 2020; Zeng et al. 2024). Slow wave potentials (SWPs), a major type of long-distance electrical signaling in higher plants, are formed by rapid *E*_m_ depolarization followed by slow repolarization. They can be triggered by touch or mechanical damage (eg damage by herbivore feeding) (Farmer et al. 2020). Mechanical crushing of a local leaf (eg leaf 8) in 5- to 6-wk-old *Arabidopsis thaliana* rosette triggers vascular leaf-to-leaf SWP propagation to distal leaves (eg leaf 13). This systemic signal subsequently induces the expression of jasmonate (JA) pathway marker gene *Jasmonate-ZIM-domain protein 10* (*JAZ10*) and promotes JA biosynthesis in both locally wounded and distal leaves, thereby supporting defense against herbivores (Mousavi et al. 2013; Kumari et al. 2019).

In Arabidopsis, plasma membrane Autoinhibited H^+^-ATPases 1 (AHA1) has been identified as a key regulator of this process. Mutation in *AHA1* reduces wound-induced SWP depolarization amplitude and prolongs subsequent repolarization duration in *aha1-7* mutant. In addition, the *aha1-7* mutant displays enhanced *JAZ10* induction, higher JA and JA-isoleucine (JA-Ile) accumulation, consequently reducing *Spodoptera littoralis* larvae weight when feeding on *aha1-7* mutant compared to wild-type plants, suggesting that AHA1 constrains systemic wound-induced JA signaling and antiherbivory defense (Kumari et al. 2019). In addition, SWP propagation also depends on the glutamate-activated, calcium (Ca^2+^)-permeable channels Glutamate Receptor-Like 3.3 (GLR3.3) and GLR3.6 in vascular tissue. Notably, GLR3.3/3.6 require an alkaline apoplastic pH for their activation (Mousavi et al. 2013). This pH sensitivity supports a model in which wound-induced inhibition of plasma membrane AHA1 facilitates apoplast alkalinization, which cooperates with glutamate release into the apoplast after wounding, activates GLRs, and facilitates long-distance electrical and Ca^2+^ signaling propagation between leaves (Shao et al. 2020). These coordinated activities of AHA1 and GLR3.3/GLR3.6 shape SWPs, downstream JA-defensive pathway, and support herbivory defense (Mousavi et al. 2013; Toyota et al. 2018; Shao et al. 2020). Similar to its structurally characterized homolog AHA2, AHA1 possesses a large (105 amino acids [aa]) C-terminal autoinhibitory domain with multiple phosphorylation sites (eg Thr^881^ and Thr^948^) (Pedersen et al. 2007; Zeng et al. 2024; Takase et al. 2026). However, whether and how this regulatory domain contributes to systemic wound signaling was unknown. This question is particularly relevant because the constitutively active AHA1 allele, *ost2-2D,* increases wound-induced SWP depolarization amplitude, shortens the repolarization phase, and suppresses *JAZ10* expression, demonstrating that the regulation of AHA1 activity is central to systemic defense signaling (Kumari et al. 2019; Shao et al. 2020).

In a recent study published in *Plant Physiology*, Pawar et al. (2026) reported that the autoinhibitory C-terminal domain of AHA1 regulates its activity and shapes phloem-associated systemic wound-induced electrical signaling, JA-pathway activation, and herbivory resistance. Pawar et al. (2026) first used *aha1-7* mutant and a native promoter *AHA1* complementation line (*AHA1c*) to reproduce previous findings on AHA1-dependent wounding-induced SWPs. Moreover, a phloem-specific complementation of *AHA1* in *aha1-7* mutant under *GLR3.3* promoter restored SWP duration and *JAZ10* induction towards wild-type levels (Pawar et al. 2026). These findings suggest that AHA1 activity in phloem-associated cells is sufficient to support leaf-to-leaf wounding signaling, consistent with the role of GLR3.3 in long-distance electrical signal propagation through phloem sieve elements (Mousavi et al. 2013; Toyota et al. 2018).

Notably, truncation of the AHA1 C-terminus generated a de-regulated variant AHA1ΔC. Expression of *AHA1ΔC* driven by its native promoter in *aha1-7* (*AHA1ΔC*) showed enhanced H^+^ pumping activity across the plasma membrane and a shortened SWP repolarization phase. Consequently, *Spodoptera frugiperda* larvae gained more weight on *AHA1ΔC* lines than on wild-type plants (Fig. 1) (Pawar et al. 2026). Consistent with this phloem-specific role, *pSUC2*-driven expression of stabilized *Small Auxin Up-RNA 19* (*SAUR19*)—which activates plasma membrane AHA by inhibiting clade-D type 2C protein phosphatases also increased larvae weight gain, whereas *Lipoxygenase 6* (*LOX6*) promoter–driven *SAUR19* expression in xylem-associated cells had minimum impact. Taken together, these results indicated that C-terminal dependent AHA1 activity in phloem-associated cells coordinates SWP duration, JA pathway, and herbivory resistance (Fig. 1) (Pawar et al. 2026).

**Figure 1:**
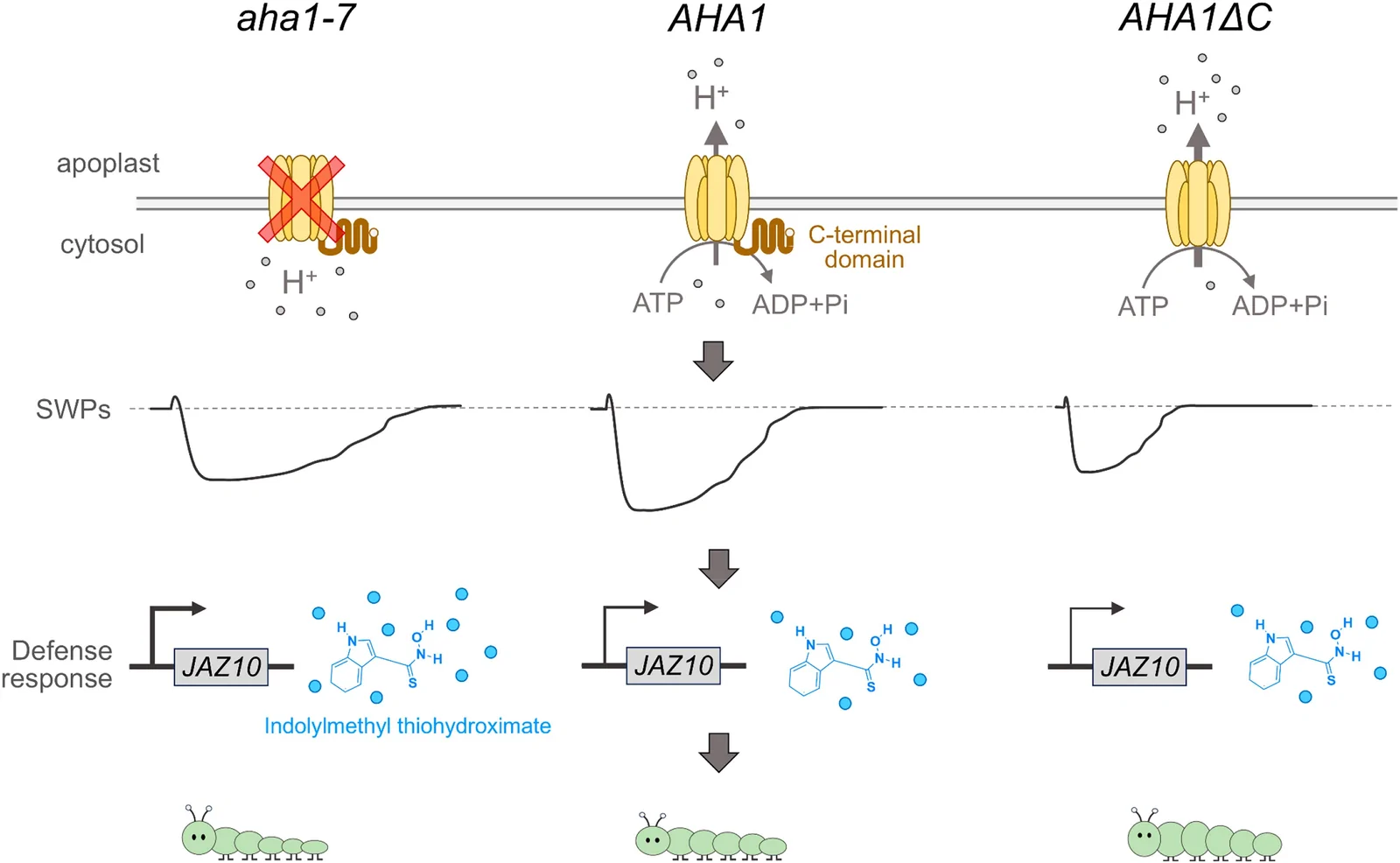
A schematic model comparing AHA1-dependent regulation of wound-induced signaling in *aha1-7*, (wild-type) *AHA1*, and *AHA1ΔC* plants. The figure shows differences in plasma membrane H^+^-ATPase activity, slow wave potentials (SWPs) propagation, defense gene *JAZ* activation, glucosinolate (GS)-biosynthetic intermediate indolylmethyl thiohydroximate accumulation, and herbivore resistance among the 3 *Arabidopsis* genotypes. In wild-type plants (middle panel), AHA1-mediated H^+^-extrusion contributes to wounding-stimulated vascular SWP propagation and subsequent activation of JA-marker *JAZ10* and GS-associated metabolic responses, including accumulation of indolylmethyl thiohydroximate. Together, these responses support antiherbivore defense (Pawar et al. 2026). In the *aha1-7* mutant (left panel), loss of *AHA1* reduces the SWP depolarization amplitude and prolongs its repolarization duration. Despite this, *aha1-7* mutant exhibits enhanced *JAZ10* induction and greater GS-biosynthetic intermediate accumulation thereby restricting both *S. littoralis* and *S. frugiperda* larval growth and enhancing herbivore resistance (Kumari et al. 2019; Pawar et al. 2026). In right panel, expression of C-terminal truncated *AHA1* (*AHA1ΔC)* by native *AHA1* promoter relieves autoinhibition, which increases plasma membrane H^+^ pumping activity and shortens SWP repolarization phase (Pawar et al. 2026), phenocopying the constitutively active *ost2-2D* allele (Kumari et al. 2019). This native *AHA1* promoter complementation does not alter GS-biosynthetic intermediate in distal leaves. Expression of *AHA1ΔC* in *GLR3.3*-expressing phloem-associated cells reduces *JAZ10* activation, compared to wild-type plants (Pawar et al. 2026), similar to that in *ost2-2D* allele (Kumari et al. 2019). Together, constitutively active AHA1ΔC leads to greater *S. frugiperda* larval weight gain. Thus, C-terminal autoinhibition of AHA1 coordinates SWP dynamics with JA-dependent and metabolic defense outputs during systemic wound signaling (Pawar et al. 2026). Chemical structure of indolylmethyl thiohydroximate was generated by ChemDraw (2023) on the basis of chemical compound information available on PubChem (https://pubchem.ncbi.nlm.nih.gov/). Adapted from Kumari et al. (2019) and Pawar et al. (2026).

Untargeted metabolomic analysis revealed hundreds of differentially accumulated metabolites in both *aha1-7* and *AHA1ΔC* lines under normal and wounding conditions, compared to wild-type plants. Close inspection identified a glucosinolate-biosynthetic intermediate, indolylmethyl thiohydroximate, strongly accumulated in both locally wounded and distal leaves of *aha1-7* mutant (Fig. 1) (Pawar et al. 2026). This intermediate only accumulated in locally wounded leaves in the *AHA1ΔC* line. These results link AHA1-dependent systemic wound signaling with defensive glucosinolate-related metabolic reprogramming (Farmer et al. 2020; Pawar et al. 2026).

This study reveals that the AHA1 C-terminal region functions as a key regulatory module in sieve elements to control the duration of the SWPs, ensuring effective propagation of wound-induced signaling to systemic leaves and optimizing plants’ defense responses against herbivores. Nevertheless, this study did not identify the upstream regulatory proteins that act on the AHA1 C-terminal domain during systemic wound signaling (Pawar et al. 2026). Takase et al. (2026) has recently demonstrated a novel AHA1 regulatory module in which C5 Raf-like protein kinase Raf36 forms a heterocomplex with C7 Raf-like kinase CBC1. Raf36–CBC1 interaction directly phosphorylates AHA1 at C-terminal Thr^881^ residue to stimulate H^+^ pumping (Takase et al. 2026). Several observations support Raf36 as a potential upstream regulator of AHA1 during wounding responses: (i) Raf36-depdenent Thr^881^ phosphorylation promotes H^+^-ATPase activity and plant growth; consistently, both *aha1-7* and *raf36-1* mutants exhibit reduced growth, while expression of the phosphomimetic AHA1^T881D^ variant restores the growth defect of *raf36-1* (Kumari et al. 2019; Takase et al. 2026); (ii) AHA1, together with AHA3, contribute to touch-induced electrical signaling in leaf veins (Yang et al. 2023); and (iii) Raf36, also named WInd-Related Kinase 1 (WIRK1), participates in wind- and touch-induced mechanosensing response (Yang et al. 2024). Together, these findings suggest that the Raf36–CBC1 module may act upstream of AHA1 during a systemic mechanosensing response. However, whether this module functions in wounding/touching/herbivory in phloem-associated cells, or regulates AHA1-dependent SWPs, remains to be determined.

## Data Availability

No new data were generated or analysed in support of this research.
